# Transmission characteristics and inactivated vaccine effectiveness against transmission of the SARS-CoV-2 Omicron BA.2 variant in Shenzhen, China

**DOI:** 10.3389/fimmu.2023.1290279

**Published:** 2024-01-08

**Authors:** Xiaofeng He, Yuxue Liao, Yuanhao Liang, Jiexin Yu, Wei Gao, Jia Wan, Yi Liao, Jiao Su, Xuan Zou, Shixing Tang

**Affiliations:** ^1^ Department of Epidemiology, School of Public Health, Southern Medical University, Guangzhou, China; ^2^ Institute of Evidence-Based Medicine, Heping Hospital Affiliated to Changzhi Medical College, Changzhi, China; ^3^ Office of Emergency, Shenzhen Center for Disease Control and Prevention, Shenzhen, China; ^4^ Third Class of 2019 of Clinical Medicine, Suzhou Medical College, Soochow University, Suzhou, Jiangsu, China; ^5^ Department of Biochemistry, Changzhi Medical College, Changzhi, China; ^6^ Department of Infectious Diseases, Nanfang Hospital, Southern Medical University, Guangzhou, China

**Keywords:** COVID-19, vaccines, effectiveness, Omicron (B.1.1.529), BA.2 subvariant, transmission

## Abstract

We conducted a retrospective cohort study to evaluate the transmission risk of severe acute respiratory syndrome coronavirus 2 (SARS-CoV-2) Omicron BA.2 variant and the effectiveness of inactivated COVID-19 vaccine boosters in Shenzhen during a BA.2 outbreak period from 1 February to 21 April 2022. A total of 1,248 individuals were infected with the BA.2 variant, and 7,855 close contacts were carefully investigated. The risk factors for the high secondary attack rate of SARS-CoV-2 infection were household contacts [adjusted odds ratio (aOR): 1.748; 95% confidence interval (CI): 1.448, 2.110], younger individuals aged 0–17 years (aOR: 2.730; 95% CI: 2.118, 3.518), older persons aged ≥60 years (aOR: 1.342; 95% CI: 1.135, 1.588), women (aOR: 1.442; 95% CI: 1.210, 1.718), and the subjects exposed to the post-onset index cases (aOR: 8.546; 95% CI: 6.610, 11.050), respectively. Compared with the unvaccinated and partially vaccinated individuals, a relatively low risk of secondary attack was found for the individuals who received booster vaccination (aOR: 0.871; 95% CI: 0.761, 0.997). Moreover, a high transmission risk was found for the index cases aged ≥60 years (aOR: 1.359; 95% CI: 1.132, 1.632), whereas a relatively low transmission risk was observed for the index cases who received full vaccination (aOR: 0.642; 95% CI: 0.490, 0.841) and booster vaccination (aOR: 0.676; 95% CI: 0.594, 0.770). Compared with full vaccination, booster vaccination of inactivated COVID-19 vaccine showed an effectiveness of 24.0% (95% CI: 7.0%, 37.9%) against BA.2 transmission for the adults ≥18 years and 93.7% (95% CI: 72.4%, 98.6%) for the adults ≥60 years, whereas the effectiveness was 51.0% (95% CI: 21.9%, 69.3%) for the individuals of 14 days to 179 days after booster vaccination and 51.2% (95% CI: 37.5%, 61.9%) for the non-household contacts. The estimated mean values of the generation interval, serial interval, incubation period, latent period, and viral shedding period were 2.7 days, 3.2 days, 2.4 days, 2.1 days, and 17.9 days, respectively. In summary, our results confirmed that the main transmission route of Omicron BA.2 subvariant was household contact, and booster vaccination of the inactivated vaccines was relatively effective against BA.2 subvariant transmission in older people.

## Introduction

The severe acute respiratory syndrome coronavirus 2 (SARS-CoV-2) Omicron (B.1.1.529) variant was first identified in South Africa on 24 November 2021; it was categorized as a variant of concern on 26 November 2021, by the World Health Organization (1). In January 2022, over 98% of new infections were caused by the Omicron variant worldwide (2). Moreover, the Omicron variant had over 50 mutations and 26–35 amino acids on the spike protein that differed from all previous variants (3). Furthermore, Omicron had stronger transmissibility than other variants, making infection more difficult to control (4–6). Between February and April 2022, an Omicron BA.2 sub-lineage outbreak occurred in Shenzhen, Guangdong, China.

It is crucial to understand the transmission characteristics of the Omicron BA.2 sub-lineage in order to develop strategies to prevent future epidemic or outbreaks. Moreover, a clearer understanding of the Omicron BA.2 sub-lineage will aid in understanding the time-interval distribution of the crucial spreading events. For instance, knowing the incubation period or the time from infection to clinical symptoms can help us determine the necessary durations of isolation and quarantine periods for the close contacts (7). In addition, the generation interval (GI) or the time from index case infection to secondary attack infection (SAI) may provide insight into the measures needed to track close contacts (8). Therefore, it is essential to analyze the distribution of the time intervals of the key spreading events for the Omicron BA.2 sub-lineage (9, 10).

Several studies have reported the vaccine effectiveness (VE) of inactivated COVID-19 vaccines against Omicron BA.2 with regard to various infection outcomes in China (11–15). Although one study evaluated VE against Omicron BA.5.2 sub-lineage transmission in China (16), more studies are definitely needed to answer the questions about the extent to which the inactivated vaccines are related to the risk of transmission (17). In this study, we conducted a retrospective cohort study to evaluate the characteristics, dynamics, and risk of transmission of SARS-CoV-2 Omicron variant infection, to estimate the effectiveness of inactivated vaccine boosters against transmission in Shenzhen during an Omicron BA.2 sub-lineage outbreak period from 1 February to 21 April 2022.

## Methods

### Study setting and design

This study was conducted as a retrospective cohort study and included all individuals with laboratory-confirmed infections and their close contacts between 1 February and 21 April 2022, in Shenzhen, Guangdong, China, during an Omicron BA.2 sub-lineage outbreak period. This report was developed using the S1 STROBE Checklist. The first Omicron BA.2–infected individual was identified on 1 February 2022, in Shenzhen, after which the variant quickly spread among the population. In response to the epidemic of COVID-19, including Omicron infection, very stringent preventive measures were quickly implemented and included by the local government (1): identification and isolation of SARS-CoV-2–infected subjects and their close contacts. All the infected patients were asked to stay and treated in the hospitals of infectious diseases, whereas all the close contacts (i.e., household and non-household) were exclusively quarantined in the specific facilities to receive medical observation and screening for SARS-CoV-2 every 2–3 days by reverse transcription polymerase chain reaction (RT-PCR) tests for SARS-CoV-2 to monitor whether they were infected with SARS-CoV-2 (2); in the areas of COVID-19 epidemic, people were asked to stay in the home and screen for SARS-CoV-2 every 2–3 days to identify any potential SARS-CoV-2 infection due to potential delays in viral load reaching detectable levels; and (3) when necessary, some districts or even the whole city may be locked down to prevent the expansion of COVID-19 epidemic. These measures influenced the transmission and resulted in lower secondary attack rate of SARS-CoV-2 BA.2 infection.

### SARS-CoV-2 infections

Data on Omicron BA.2 sub-lineage–infected individuals and their close contacts were retrospectively collected between 1 February and 21 April 2022, from Shenzhen Center for Disease Control and Prevention in China. For individuals with BA.2 sub-lineage infection, we extracted data, which mainly included age, sex, history of exposure, contact setting, onset date of clinical symptoms, first positive test date, serial PCR test results, and history of COVID-19 vaccination. In this study, SARS-CoV-2 infection was indicated by a positive nucleic acid amplification test regardless of illness severity. Asymptomatic infection referred to a positive nucleic acid amplification test without clinical symptoms (12). Symptomatic COVID-19 was defined as PCR-confirmed infection with any COVID-19 clinical symptom. COVID-19 pneumonia was diagnosed on the basis of characteristics of chest computed tomography imaging.

### Close contacts and transmission pairs

**Figure 1:**
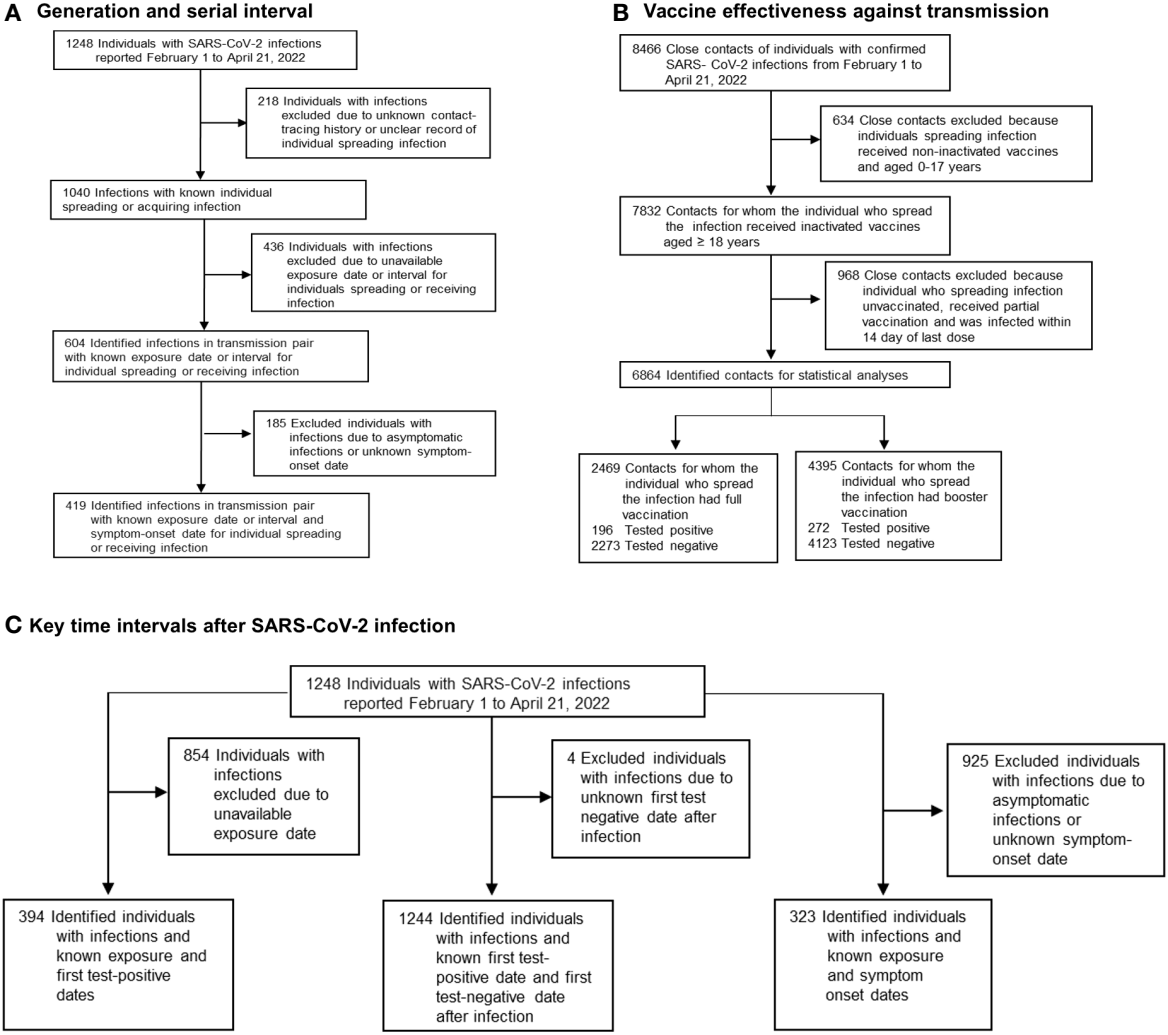
Flowchart of sample selection in this study. **(A)** The sample selection procedure for transmission pairs that were used for estimating generation and serial interval is presented. **(B)** The sample selection procedure for eligible close contacts who were used for estimating vaccine effectiveness against transmission is presented. **(C)** The sample selection procedure for eligible SARS-CoV-2 infections that were used for estimating the period from exposure to viral shedding, viral shedding period, and incubation period is presented.

Close contacts were individuals in the same exposure settings within proximity of a COVID-19–infected individual without any effective protection (18). Exposure settings for close contacts included household and non-household settings (19). In this study, individuals at risk of exposure were considered to be close contacts of confirmed cases. Those close contacts who eventually tested positive for COVID-19 were treated as infected individuals (infectees) and their index cases (who were originally confirmed to have COVID-19) as infectors. We extracted these epidemiologically linked infectees and infector pairs and their individual data for all transmission pairs. **Figure 1** shows a flowchart of the sample selection procedure for transmission pairs.

### Vaccination status

We classified the individuals infected with the BA.2 sub-lineage and their close contacts into an unvaccinated group, a partially vaccinated group, a fully vaccinated group, and a booster-vaccinated group based on their electronic COVID-19 vaccination records. The unvaccinated group had received no COVID-19 vaccines before their last known contact with a confirmed infected individual or COVID-19 onset time. The partial vaccination group included those who had their first dose of viral vector (non-replicating) COVID-19 vaccines <14 days, their first dose of inactivated COVID-19 vaccine ≥0 days or their second dose of inactivated COVID-19 vaccine <14 days, or their first and second doses of protein subunit COVID-19 vaccines ≥0 days or third dose of protein subunit COVID-19 vaccine <14 days before their last known contact or symptom onset time. Full vaccination included those ≥14 days after their first dose of viral vector (non-replicating) COVID-19 vaccine, ≥14 days after their second dose of inactivated COVID-19 vaccine, ≥14 days after their third dose of protein subunit COVID-19 vaccine, and <7 days after booster vaccination before their last known contact or onset time. The booster vaccination group included those ≥7 days after their second dose of viral vector (non-replicating) COVID-19 vaccine and ≥7 days after their third dose of inactivated COVID-19 vaccine or other third dose of heterologous COVID-19 vaccine before their last known contact or onset time. To evaluate the inactivated COVID-19 VE against transmission, the full vaccination group included only those ≥14 days after their second dose, excluding those 0–7 days after their third dose, and the booster vaccination group included only those ≥14 days after their third dose because index cases who received their booster vaccination within 7–13 days were absent in this study.

### Statistical analysis

Baseline characteristics were calculated as the number (%) for categorical variables and the median [interquartile range (IQR)] for continuous variables. Differences in proportions and median values were analyzed with the chi-squared and Wilcoxon tests, respectively. The secondary attack rate (7, 20) was estimated by dividing the number of individuals with secondary infections by the overall number of close contacts related to index cases. Moreover, we analyzed the proportion of cases of supercritical transmission (SCT), which referred to cases with an individual transmission number ≥1 (21, 22).

To assess the risk of SAI with the Omicron BA.2 sub-lineage, a stepwise logistic regression model was used involving age, age of index case, sex, sex of index case, contact setting, type of index case, COVID-19 vaccination status of close contacts, COVID-19 vaccination status of index case, and exposure to index case before or after onset. Of the index cases in this study, most were in the groups with full and booster vaccinations with inactivated COVID-19 vaccines. Therefore, we focused on evaluating the effectiveness of inactivated vaccine boosters against transmission compared with that of full vaccination. The crude odds ratio (OR) was calculated by applying a univariate logistic regression model, and the adjusted OR (aOR) was calculated using a multivariable ordinary logistic regression model by adjusting for potential confounding variables, including sex of index case and their close contacts, age of index case and their close contacts, contact setting, COVID-19 vaccination status of close contacts, and exposure to an index case before or after onset. The crude or adjusted VE (aVE) was calculated as (1 − OR) × 100%, and the OR is the odds ratio for the incidence of secondary infection (23–25). Subgroups were evaluated on the basis of age, time interval of COVID-19 vaccination, and contact setting. All statistical analyses were conducted with IBM SPSS Statistics 25.0.

Then, the time intervals between key events were estimated involving GI, serial interval (SI), latent period, viral shedding period, and incubation period with gamma distributions using a Bayesian framework (16). All of these analyses were performed with the rstan package in R software (R Foundation for Statistical Computing, Vienna, Austria). GI was estimated as the mean duration between the time of SAI with known exposure time and time of infection of index case. SI was estimated as the mean duration between the onset time of clinical symptoms in index cases and the onset time of clinical symptoms in secondary cases generated by those index cases. Moreover, we estimated the distribution of the latent period, from exposure to viral shedding, using the first positive test date as an alternative to the time of viral shedding; we included cases with a known exposure date or a first positive PCR test date. Furthermore, we estimated the viral shedding period using the first positive PCR test date and the first negative test date as the end date of viral shedding. Finally, the incubation period was estimated using symptomatic cases with a known exposure date. Subgroup analyses were conducted according to contact setting, age, symptom status, and vaccination status. We estimated the daily instantaneous reproduction number (Rt) with the EpiEstim package (7) in R software.

## Results

In this study, we identified 7,855 uninfected close contacts {4,822 men [61.4%]; mean age, 36.1 [standard deviation (SD): 13.0] years} and 1,248 individuals infected with Omicron BA.2 [710 men (56.9%); mean age, 34.4 (SD: 16.5) years]. Of the infected individuals, 203 (16.3%) were asymptomatic and 1,045 (83.7%) were symptomatic (**Supplementary Table 1**). Among the 1,045 symptomatic cases, 975 (93.3%) presented with mild symptoms and 70 (6.7%) had COVID-19 pneumonia (**Supplementary Table 1**). The most frequently reported symptoms were pyrexia [573 (60.5%) of 947], cough [341 (36.0%)], pharyngeal pain [223 (23.5%)], and fatigue [135 (14.3%)] (**Supplementary Table 2** and **Supplementary Figure 1**). The number of daily infections continuously increased until reaching a peak of 107 infections on 15 March 2022. Thereafter, the number of daily infections gradually decreased to one infection on 21 April 2022 (**Supplementary Figure 2**).

### Characteristics of close contacts and index cases

We included 8,466 close contacts associated with 644 index cases. Among the 644 index cases, 368 (57.1%) were men, and the median age was 35.0 years (IQR: 27.0, 47.0), with 545 (84.6%) index cases aged between 20 years and 59 years (**Table 1**). The majority of index cases had mild COVID-19 (510, 79.2%) or asymptomatic infection (101, 15.7%), whereas the remaining cases had COVID-19 pneumonia (33, 5.1%) (**Table 1**). Among the index cases, there were 66 (10.3%), 29 (4.5%), 232 (36.0%), and 317 (49.2%) cases in the unvaccinated, partially vaccinated, fully vaccinated, and booster-vaccinated groups, respectively (**Table 1**). We also found that the cycle threshold (Ct) values for the first positive test were 24.0 (IQR: 20.0, 29.0) and 23.0 (IQR: 18.0, 28.0) for the ORF1ab and N genes, respectively (**Table 1**). Finally, significant differences (*P* < 0.05) were observed for age and Ct value of the first positive test for the ORF1ab or N gene between the transmitted index cases (216, 33.5%) and non-transmitted index cases (428, 66.5%) (**Table 1**).

**Table 1:** Characteristics of index cases, Shenzhen, China, February to April 2022.

Characteristics of index cases	Non-transmitted index cases [428 (66.5)]	Transmitted index cases [216 (33.5)]	Overall index cases (644)	*P*
Gender				
Male	249 (58.2)	119 (55.1)	368 (57.1)	0.423
Female	179 (41.8)	97 (44.9)	276 (42.9)	
Age, years				
Overall (median, IQR)	34.0 (27.3, 47.0)	38.0 (26.0, 48.8)	35.0 (27.0, 47.0)	0.757
0–9	14 (3.2)	17 (7.9)	31 (4.8)	0.019
10–19	24 (5.6)	11 (5.1)	35 (5.5)	
20–29	96 (22.4)	44 (20.4)	140 (21.7)	
30–39	130 (30.4)	48 (22.2)	178 (27.6)	
40–49	76 (17.8)	48 (22.2)	124 (19.3)	
50–59	71 (16.6)	32 (14.8)	103 (16.0)	
≥ 60	17 (4.0)	16 (7.4)	33 (5.1)	
COVID-19 vaccine dose				
None	38 (8.9)	28 (13.0)	66 (10.3)	0.119
Partial vaccination	19 (4.4)	10 (4.6)	29 (4.5)	
Full vaccination	147 (34.4)	85 (39.4)	232 (36.0)	
Booster vaccination	224 (52.3)	93 (43.0)	317 (49.2)	
Type of index cases				
Asymptomatic	66 (15.4)	35 (16.2)	101 (15.7)	0.900
Mild COVID-19	341 (79.7)	169 (78.2)	510 (79.2)	
COVID-19 pneumonia	21 (4.9)	12 (5.6)	33 (5.1)	
Ct value of the first positive test for the ORF1ab				
Overall (median, IQR)	24.0 (20.0, 30.0)	23.0 (19.0, 27.0)	24.0 (20.0, 29.0)	0.003
Ct value of the first positive test for the N gene				
Overall (median, IQR)	23.0 (19.0, 28.5)	22.0 (18.0, 26.0)	23.0 (18.0, 28.0)	0.007

Data are n (%), unless otherwise specified. *None: not vaccinated; part vaccination: <14 days after first vaccination for viral vector (non-replicating) vaccine, after first vaccination or <14 days after second vaccination for COVID-19 inactivated virus vaccine, and after first and second vaccination or <14 days after third vaccination COVID-19 protein subunit vaccine (if any); full vaccination: ≥14 days after first vaccination for viral vector (non-replicating) vaccine, ≥14 days after second vaccination for COVID-19 inactivated virus vaccine, ≥14 days after third vaccination for COVID-19 protein subunit vaccine, and <7 days after booster vaccination (if any); booster vaccination: ≥7 days after second dose for COVID-19 viral vector (non-replicating) vaccines or ≥7 days after third dose for COVID-19 any vaccine [including protein subunit, inactivated virus, and viral vector (non-replicating) vaccines] (if any).

Among the 8,466 close contacts, 5,150 (52.8%) were men, with a median age of 34.0 years (IQR: 27.0, 45.0 years) and 7,402 (87.4%) individuals aged between 20 years and 59 years (**Table 2**). In terms of contact setting, there were 1,568 (18.5%) and 6,898 (81.5%) household and non-household settings, respectively (**Table 2**). Contact frequency was categorized as “occasionally or sometimes” for 7,258 (85.7%) individuals and “frequently” for 1,208 (14.3%) (**Table 2**). Moreover, 737 (8.7%) close contacts did not receive any COVID-19 vaccines, and the percentages in the partial, full, and booster vaccination groups were 3.3%, 36.8%, and 51.1%, respectively (**Table 2**). Furthermore, of the 8,466 close contacts, 611 {7.2% [95% confidence interval (CI): 6.7%, 7.8%]} were secondarily infected, 85 [1.0% (95% CI: 0.8%, 1.2%)] were asymptomatic, 499 [5.9% (95% CI: 5.4%, 6.4%)] had mild COVID-19, and 27 [0.3% (95% CI: 0.2% to 0.5%)] had COVID-19 pneumonia. Of these 611 cases, 85 (13.9%) presented with no clinical symptoms and 526 (86.1%) presented with clinical symptoms (**Table 2**). Finally, significant differences (*P* < 0.05) were found for age, gender, contact setting, contact frequency, and COVID-19 vaccination status between the SAI and non-infected groups (**Table 2**).

**Table 2:** Characteristics of close contacts of index cases, Shenzhen, China, February to April 2022.

Characteristics of close contacts	Non-infection [7,855 (92.8)]	SAI [611 (7.2)]	Close contacts (8,466)	*P*
Age (years)				
Overall (Median, IQR)	34.0 (27.0, 44.0)	35.0 (24.0, 49.0)	34.0 (27.0, 45.0)	
0–9	259 (3.3)	75 (12.3)	334 (4.0)	< 0.001
10–19	321 (4.1)	44 (7.2)	365 (4.3)	
20–29	2,082 (26.5)	93 (15.2)	2,175 (25.7)	
30–39	2,483 (31.6)	143 (23.4)	2,626 (31.0)	
40–49	1,450 (18.5)	115 (18.8)	1,565 (18.5)	
50–59	944 (12.0)	92 (15.1)	1,036 (12.2)	
≥ 60	316 (4.0)	49 (8.0)	365 (4.3)	
Gender				
Male	4,822 (61.4)	328 (53.7)	5,150 (60.8)	< 0.001
Female	3,033 (38.6)	283 (46.3)	3,316 (39.2)	
Contact setting				
Household	1,321 (16.8)	247 (40.4)	1,568 (18.5)	< 0.001
Non-household	6,534 (83.2)	364 (59.6)	6,898 (81.5)	
Contact frequency				
Occasionally or sometimes	6,877 (87.5)	381 (62.4)	7,258 (85.7)	< 0.001
Frequently	978 (12.5)	230 (37.6)	1,208 (14.3)	
COVID-19 vaccine dose*				
None	651 (8.3)	86 (14.1)	737 (8.7)	< 0.001
Part vaccination	262 (3.3)	21 (3.4)	283 (3.3)	
Full vaccination	2,875 (36.6)	243 (39.8)	3,118 (36.8)	
Booster vaccination	4067 (51.8)	261 (42.7)	4,328 (51.2)	
COVID-19 cases				
Asymptomatic	NA	85 (13.9)	NA	NA
Symptomatic	NA	526 (86.1)	NA	
Mild	NA	499 (81.7)	NA	
pneumonia	NA	27 (4.4)	NA	

Data are n (%), unless otherwise specified. *None: not vaccinated; part vaccination: <14 days after first vaccination for viral vector (non-replicating) vaccine, after first vaccination or <14 days after second vaccination for COVID-19 inactivated virus vaccine, and after first and second vaccination or <14 days after third vaccination COVID-19 protein subunit vaccine (if any); full vaccination: ≥14 days after first vaccination for viral vector (non-replicating) vaccine, ≥14 days after second vaccination for COVID-19 inactivated virus vaccine, ≥14 days after third vaccination for COVID-19 protein subunit vaccine, and <7 days after booster vaccination (if any); booster vaccination: ≥7 days after second dose for COVID-19 viral vector (non-replicating) vaccines or ≥7 days after third dose for COVID-19 any vaccine [including protein subunit, inactivated virus, and viral vector (non-replicating) vaccines] (if any). SAI, secondary attack infection.

### Incidence of SAI and factors associated with transmission risk

Of the 8,466 close contacts, 611 were SAI cases caused by Omicron BA.2, with an incidence of 7.2% (95% CI: 6.7%, 7.8%) (**Table 3**). There were two secondary attack and spreading infection peaks in the groups of individuals aged 0–9 years old and ≥60 years old (**Supplementary Figure 3**). Moreover, the SCT rate was higher for individuals spreading infection among the group of individuals aged ≥60 years old [26.8% (95% CI: 15.7%, 41.9%)] (**Supplementary Table 3**).

**Table 3:** Estimating the association of demographic and behavioral factors with the risk of acquiring and transmitting SARS-CoV-2 Omicron BA.2.

Characteristics	Close contacts	Secondary Cases	SAR [%, (95% CI)]	Crude OR (95% CI)	*P-*value	Adjusted OR (95% CI)	*P*-value
Age (years)							
0–17	608	110	18.1 (15.2, 21.4)	3.441 (2.741, 4.319)	< 0.001	2.730 (2.118, 3.518)	< 0.001
18–59	7493	452	6.0 (5.5, 6.6)	1 (ref)	.	1 (ref)	.
≥ 60	365	49	13.4 (10.3, 17.3)	1.554 (1.327, 1.820)	< 0.001	1.342 (1.135, 1.588)	< 0.001
Age of index cases (years)							
0–17	442	47	10.6 (8.1, 13.9)	1.656 (1.208, 2.270)	0.002	1.079 (0.761, 1.531)	0.670
18–59	7757	520	6.7 (6.2, 7.3)	1 (ref)	.	1 (ref)	.
≥ 60	267	44	16.5 (12.5, 21.4)	1.657 (1.401, 1.960)	< 0.001	1.359 (1.132, 1.632)	0.001
Gender							
Male	5150	328	6.4 (5.7, 7.1)	1 (ref)	.	1 (ref)	.
Female	3316	283	8.5 (7.6, 9.5)	1.372 (1.163, 1.618)	< 0.001	1.442 (1.210, 1.718)	< 0.001
Gender of index cases							
Male	5290	370	7.0 (6.3, 7.7)	1 (ref)	.	1 (ref)	.
Female	3176	241	7.6 (6.7, 8.6)	1.092 (0.923, 1.292)	0.307	0.967 (0.808, 1.157)	0.715
Contact setting							
Household	1568	247	15.8 (14.0, 17.6)	3.356 (2.826, 3.987)	< 0.001	1.748 (1.448, 2.110)	< 0.001
Non-household	6898	364	5.3 (4.8, 5.8)	1 (ref)	.	1 (ref)	.
COVID-19 vaccine dose*							
None/partial vaccination	1020	107	10.5 (8.8, 12.5)	1 (ref)	.	1 (ref)	.
Full vaccination	3118	243	7.8 (6.9, 8.8)	0.721 (0.568, 0.916)	0.007	0.903 (0.696, 1.170)	0.439
Booster vaccination	4328	261	6.0 (5.4, 6.8)	0.740 (0.658, 0.833)	< 0.001	0.871 (0.761, 0.997)	0.045
COVID-19 vaccine dose of index cases*							
None/partial vaccination	928	102	11.0 (9.1, 13.2)	1 (ref)	.	1 (ref)	.
Full vaccination	3020	237	7.9 (6.9, 8.9)	0.690 (0.540, 0.881)	0.003	0.642 (0.490, 0.841)	0.001
Booster vaccination	4518	272	6.0 (5.4, 6.8)	0.720 (0.639, 0.812)	< 0.001	0.676 (0.594, 0.770)	< 0.001
Type of index cases							
Asymptomatic infection	1521	105	6.9 (5.7, 8.3)	1 (ref)	.	1 (ref)	.
Symptomatic COVID-19	6945	506	7.3 (6.7, 7.9)	1.060 (0.852, 1.318)	0.602	1.172 (0.929, 1.478)	0.181
Exposure to an index case at onset^&^							
Yes	4551	73	1.6 (1.3, 2.0)	1 (ref)	.	1 (ref)	.
No	3915	538	13.7 (12.7, 14.9)	9.773 (7.662, 12.530)	< 0.001	8.546 (6.610, 11.050)	< 0.001

Data are n (%), unless otherwise specified. *None: not vaccinated; partial vaccination: <14 days after first vaccination for viral vector (non-replicating) vaccine, after first vaccination or <14 days after second vaccination for COVID-19 inactivated virus vaccine, and after first and second vaccination or <14 days after third vaccination COVID-19 protein subunit vaccine (if any); full vaccination: ≥14 days after first vaccination for viral vector (non-replicating) vaccine, ≥14 days after second vaccination for COVID-19 inactivated virus vaccine, ≥14 days after third vaccination for COVID-19 protein subunit vaccine, and <7 days after booster vaccination (if any); booster vaccination: ≥7 days after second dose for COVID-19 viral vector (non-replicating) vaccines or ≥7 days after third dose for COVID-19 any vaccine [including protein subunit, inactivated virus, and viral vector (non-replicating) vaccines] (if any). Ct, cycle threshold; SAR, secondary attack rate. ^&^Close contacts were exposed to an index at the time of symptom onset of the index.

Next, we found that the close contacts aged 0–17 years old [aOR: 2.730 (95% CI: 2.118, 3.518)] and ≥60 years old [aOR: 1.342 (95% CI: 1.135, 1.588)] had a higher SAI risk than those aged 18–59 years old (**Table 3**). Moreover, we observed that female contacts had a slightly higher SAI risk (aOR: 1.442; 95% CI: 1.210, 1.718) than the male contacts (**Table 3**). Among the exposure settings, household contacts were associated with a higher risk of SAI (aOR: 1.748; 95% CI: 1.448, 2.110) versus non-household contacts (**Table 3**). A lower SAI rate was found for the close contacts who received full vaccination compared with those who were unvaccinated and partially vaccinated [7.8% (95% CI: 6.9%, 8.8%) versus 10.5% (95% CI: 8.8% to 12.5%)] (**Table 3**), but this difference was not significant according to multivariate analysis. However, a lower risk of SAI was found for the close contacts who received booster vaccination (aOR: 0.871; 95% CI: 0.761, 0.997) compared with unvaccinated and partially vaccinated close contacts (**Table 3**). Moreover, the close contacts with exposure to an index case after onset had a higher SAI risk (aOR: 8.546; 95% CI: 6.610, 11.05) compared with those exposed to an index case before onset (**Table 3**). The frequency of contact was not separately evaluated due to multicollinearity with the household contacts.

A higher transmission rate was also found for the index cases aged 0–17 years [10.6% (95% CI: 8.1%, 13.9%)] compared with those aged 18–59 years [6.7% (95% CI: 6.2%, 7.3%)] (**Table 3**). A relatively high transmission risk was found for index cases aged ≥60 years (aOR: 1.359; 95% CI: 1.132, 1.632) compared with those aged 18–59 years. In terms of COVID-19 vaccination status of index cases, a lower transmission risk was observed for index cases who received full vaccination (aOR: 0.642; 95% CI: 0.490, 0.841) and booster vaccination (aOR: 0.676; 95% CI: 0.594, 0.770) compared with those who were unvaccinated and partially vaccinated.

### VE against transmission of the Omicron BA.2 variant

Overall, 6,864 close contacts were related to 502 index cases who spread infections; their data were analyzed to assess VE of inactivated COVID-19 vaccines against BA.2 transmission for those aged ≥18 years old (**Table 4**). Of the close contacts, 2,469 (36.0%) and 4,395 close contacts (64.0%) received full and booster vaccinations, respectively. Among index cases, 184 (36.7%) and 318 (63.3%) individuals received full and booster vaccinations, respectively. Overall, the aVE was 24.0% (95% CI: 7.0%, 37.9%) against BA.2 transmission for booster vaccination compared with full vaccination. An aVE against transmission of 93.7% (95% CI: 72.4%, 98.6%) was found among those aged ≥60 years. Moreover, the aVE against transmission remained high 14 to 179 days after booster vaccination [51.0% (95% CI: 21.9%, 69.3%)]. We also found that the aVE [51.2% (95% CI: 37.5%, 61.9%)] against transmission was high for the non-household contact setting.

**Table 4:** Inactivated vaccine effectiveness of booster vaccination vs full vaccination against transmission.

Characteristic	Close contacts, no. (%)				Crude		Adjusted*	
	Individuals spreading infection with full vaccination (reference)		Individuals spreading infection with booster vaccination		OR (95% CI)	VE (95% CI), %	OR (95% CI)	VE (95% CI), %
	Contact tested positive	Contact tested negative	Contact tested positive	Contact tested negative				
Overall	196 (7.9)	2273 (92.1)	272 (6.2)	4123 (93.8)	0.765 (0.632, 0.926)	23.5 (7.4, 36.8)	0.760 (0.621, 0.930)	24.0 (7.0, 37.9)
Age of Individuals spreading infection								
18–59	165 (7.0)	2178 (93.0)	270 (6.2)	4055 (93.8)	0.879 (0.719, 1.074)	12.1 (−7.4, 28.1)	0.868 (0.702, 1.073)	13.2 (−7.3, 29.8)
≥ 60	31 (24.6)	95 (75.4)	2 (2.9)	68 (97.1)	0.090 (0.021, 0.389)	91.0 (61.1, 97.9)	0.063 (0.014, 0.276)	93.7 (72.4, 98.6)
Time since last vaccine dose for Individuals spreading infection (day)								
14–179	25 (7.7)	299 (92.3)	267 (6.3)	3973 (93.7)	0.804 (0.525, 1.231)	19.6 (−23.1, 47.5)	0.490 (0.307, 0.781)	51.0 (21.9, 69.3)
≥ 180	171 (8.0)	1974 (92.0)	5 (3.2)	150 (96.8)	0.385 (0.156, 0.951)	61.5 (4.9, 84.4)	0.817 (0.318, 2.097)	18.3 (−109.7, 95.1)
Contact setting								
Household	61 (10.9)	499 (89.1)	108 (15.5)	589 (84.5)	1.503 (1.074, 2.103)	−50.3 (−110.3, −7.4)	1.305 (0.907, 1.879)	−30.5 (−87.9, 9.3)
Non-household	135 (7.1)	1774 (92.9)	164 (4.4)	3534 (95.6)	0.610 (0.482, 0.771)	39.0 (22.9, 51.8)	0.488 (0.381, 0.625)	51.2 (37.5, 61.9)

VE, vaccine effectiveness. *Variables adjusted in the model were sex of index cases and their close contacts, age of index cases and their close contacts, contact setting, vaccination dose of close contacts, and exposure to an index case at onset for each contact.

### Estimation of time intervals between key events

**Figure 2:**
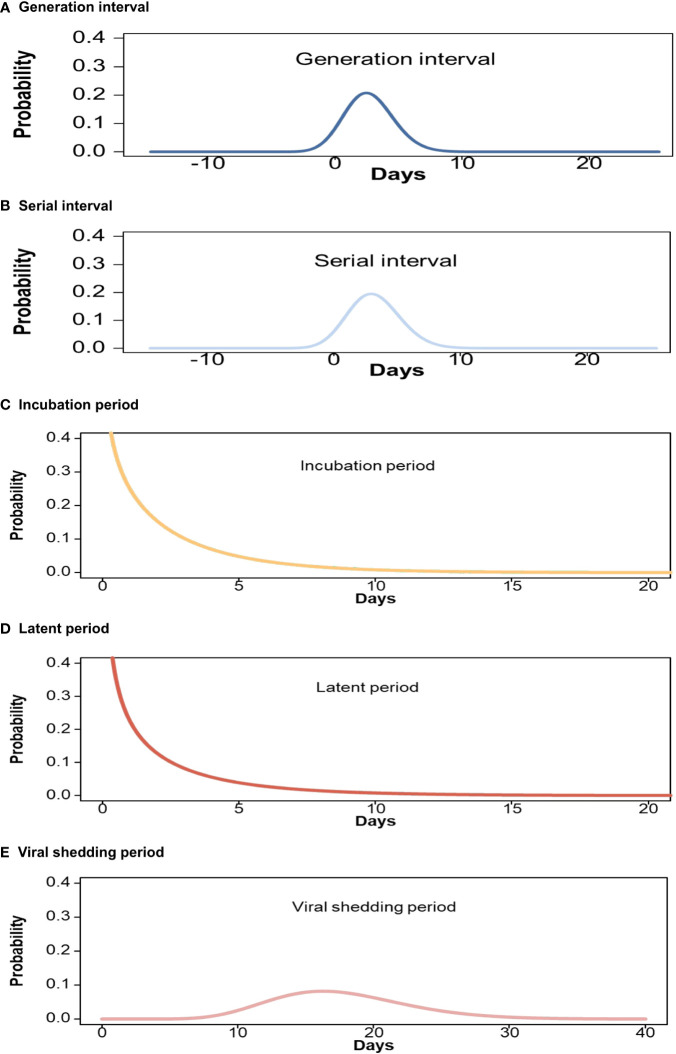
Estimated cumulative γ distributions of key time intervals for Omicron BA.2. **(A)** Generation interval; **(B)** Serial interval; **(C)** Incubation period; **(D)** Latent period; **(E)** Viral shedding period.

**Figure 3:**
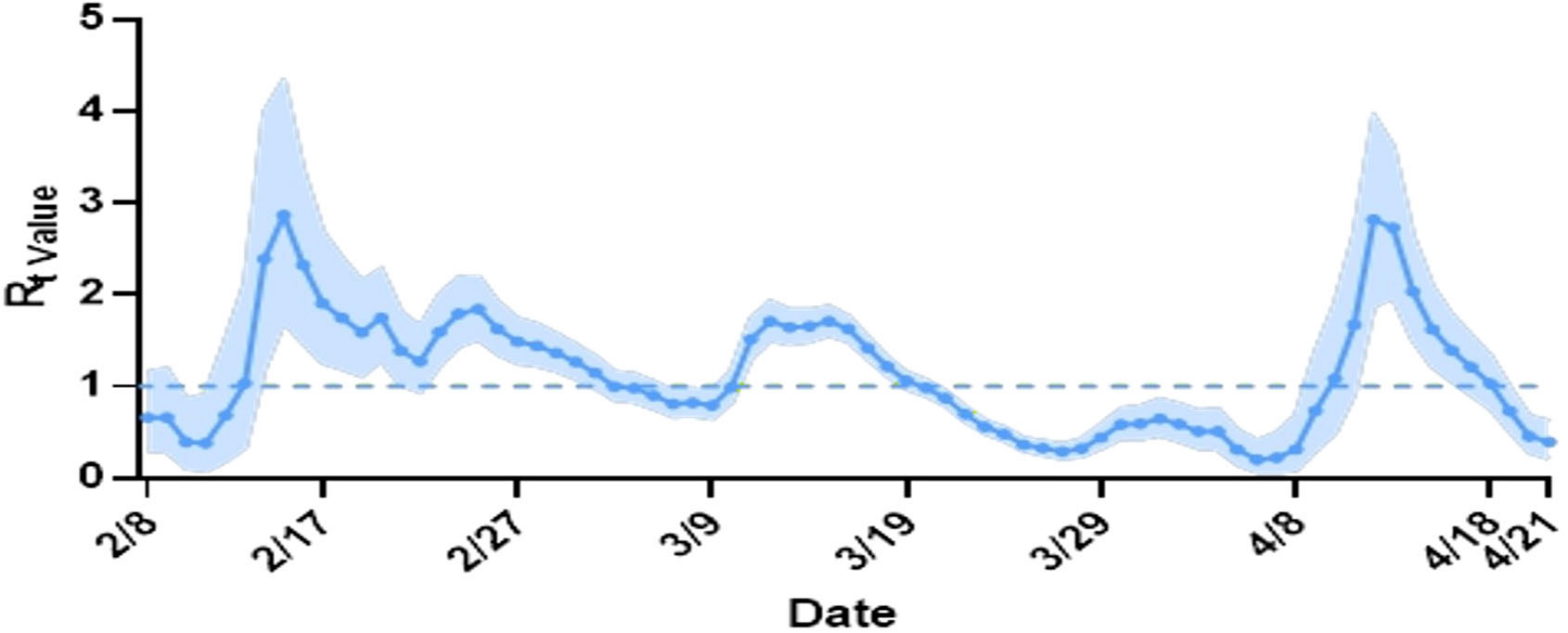
Estimated the daily instantaneous reproduction number (Rt).

We analyzed data from 604 transmission pairs to estimate the GI, which was 2.7 days [97.5% credible interval (CrI): −0.8 to 6.7 days] (**Table 5**; **Figure 2A**). In a subgroup analysis based on contact setting, the mean GI estimate was longer for non-household settings than for household settings [2.9 days (97.5% CrI: −0.8 to 7.0 days) versus 2.4 days (97.5% CrI: −0.9 to 6.0 days), respectively] (**Supplementary Table 4**). Moreover, 419 transmission pairs were used to estimate SI, which was 3.2 days (97.5% Crl: −0.6 to 7.4 days) (**Table 5**; **Figure 2B**). According to subgroup analysis based on contact setting, the mean SI estimate was similar in non-household and household settings [3.2 days (97.5% CrI: −0.7 to 7.6 days) versus 3.2 days (97.5% CrI: −0.5 to 7.2 days)] (**Supplementary Table 4**). Furthermore, 323 transmission pairs were used to estimate the mean incubation period of 2.4 days (97.5% CrI: 0.0 to 9.9 days) (**Table 5**; **Figure 2C**). Individuals aged 18–59 years had a longer mean incubation period than the overall average (**Supplementary Table 4**). The mean latent period was 2.1 days (97.5% CrI: 0.0 to 9.7 days) (**Table 5**; **Figure 2D**) based on an analysis of the data from 394 transmission pairs, although individuals aged 0–17 years had a shorter mean latent period (**Supplementary Table 4**). Finally, we found that the mean viral shedding period was 17.9 days (97.5% CrI: 9.4, 28.5 days) (**Table 5**; **Figure 2E**) according to analysis of data from 1,244 individuals with BA.2 infection. A shorter mean viral shedding period (**Supplementary Table 4**) was observed among asymptomatic individuals aged 0–17 years. The estimated reproduction number declined from 2.8 on 14 February 2022 to 0.4 on 21 April 2022 (**Figure 3**).

**Table 5:** Estimates of the time intervals about key events.

Time intervals	Sample size	Parameters [mean (SD)]	Mean (days)	Quantiles (0.025–0.975, days)
Generation interval	604	Shape = 79.19 (4.55), rate = 4.6 (0.26), shift = 14.5	2.7	−0.8, 6.7
Serial interval	419	Shape = 73.45 (5.06), rate = 4.15 (0.29), shift = 14.5	3.2	−0.6, 7.4
Latent period	394	Shape = 0.55 (0.04), rate = 0.26 (0.02)	2.1	0.0, 9.7
Incubation period	323	Shape = 0.75 (0.06), rate = 0.31 (0.03)	2.4	0.0, 9.9
Viral shedding period	1244	Shape = 12.55 (0.50), rate = 0.71 (0.03)	17.9	9.4, 28.5

## Discussion

In this study, we conducted a comprehensive analysis to evaluate the characteristics, dynamics, and risk of transmission of BA.2 infection. Moreover, we estimated the effectiveness of inactivated COVID-19 vaccine boosters against transmission in Shenzhen, China.

The secondary attack rate was 7.2% among close contacts with BA.2 infection. No significant difference was found for the transmission risk related to the type of index case or gender of index case; however, female close contacts showed a higher SAI risk. Moreover, individuals aged 0–17 years old and ≥60 years old had a higher SAI and transmission incidence compared with those aged 18–59 years old. Although this difference was not significant for the index cases aged 0–17 years old compared with those aged 18–59 years old, a higher SAI incidence was found for index cases aged 0–17 years. Furthermore, we observed that close contact exposure to an index case after onset was associated with a higher SAI risk than exposure to an index case before onset. This may be because post-onset index cases may carry a higher viral load. Moreover, household contacts had a higher SAI risk than non-household contacts. SAI incidence was 15.8% among household contacts, which was not consistent with the incidence of SAI in Denmark in a published study (21). This may be because the local government in Shenzhen immediately compulsorily isolated household contacts at designated facilities after the index cases were diagnosed, resulting in a relatively low secondary attack rate compared with published study in Denmark (21). In addition, we observed that individuals who received booster vaccination had a lower risk of SAI compared with unvaccinated or only partial vaccinated subjects although the effectiveness was not very high. Several studies have reported a lower VE against Omicron BA.2 infection than other variants of SARS-CoV-2 (26–31).

The VE against transmission is rarely reported (7, 16, 32). We found that a lower risk of SAI for close contacts was associated with index cases who received full or booster vaccinations compared with index cases in the unvaccinated and partially vaccinated groups. The results further showed the importance of increasing vaccine coverage to mitigate the spread of COVID-19 (33). We then excluded individuals who were unvaccinated or partially vaccinated because most index cases [549 of 644 individuals (85.2%)] and their close contacts [7,446 of 8,466 individuals (88.0%)] in this study had received at least two doses of COVID-19 vaccines. Moreover, individuals aged 0–17 years old were not included in the analysis of VE against transmission because they were not covered by the booster immunization campaigns in China. Furthermore, we excluded those who received non-inactivated COVID-19 vaccines because most individuals in this study had received the inactivated COVID-19 vaccines (>90%). Therefore, we focused on evaluating the inactivated VE against transmission for the individuals with booster vaccination versus full vaccination.

The overall aVE was 24.0% against BA.2 transmission for booster vaccination compared with full vaccination indicating no effective protection. Our estimated overall VE against BA.2 transmission was consistent with the previous estimates of 28.9% for Omicron BA.5.2 variant [18]. However, an aVE of 93.7% against transmission was found among those aged ≥60 years older, but not in those aged 18–59 years older when comparing the booster versus full vaccination groups. As we know, the current COVID-19 vaccines are less effective in preventing SARS-CoV-2 Omicron infection but are useful in mitigating the severity of COVID-19 especially in old adults (26–31). Because of the high frequency of complications, such as hypertension and diabetes mellitus, senior adults are more easily to suffer from severe COVID-19. Moreover, our study indicated that family members and household contacts were prone to being infected with SARS-CoV-2. Old adults are more often to stay in home and became susceptible to SARS-CoV-2 infection. We indeed observed two secondary infection peaks in children and old adults. These factors might contribute to the observed high effectiveness in the age group of ≥60 years. A moderate level of protection was observed against the transmission in non-household contact setting, whereas no significantly protective effect was found in household contact setting. Moreover, a moderate level of protection was observed against the transmission at 14 to 179 days after booster vaccination, whereas no any effectiveness was found against the transmission at ≥180 days after booster vaccination. The results indicate the protective effect was waning over time against BA.2 transmission. Wang et al. [18] observed inactivated vaccines booster immunization provided ineffective protection against BA.5.2 transmission at over 90 days after booster vaccination. As we know, SARS-CoV-2 has been evolving and even the Omicron sub-lineages are constantly emerging, such as XXB, EG.5, and HV.1 (1). The emerging variants and sub-lineages showed new features including different virulence, transmission capability and resistance to the existing neutralizing immunity, which, in turn, require the continuous assessment of VE against emerging SARS-VoV-2 variants. Furthermore, new COVID-19 vaccines including different SARS-CoV-2 variants, i.e., bivalent or trivalent COVID-19 vaccines, have been developing and implemented in response to emerging Omicron sub-lineage according to WHO recommendation. However, the VE remains to be evaluated by using real world data. Therefore, we emphasize the importance of continuously evaluating VE for emerging Omicron subvariants. Overall, these results indicated that inactivated vaccine boosters can effectively prevent the transmission of index cases, especially in the population aged ≥60 years.

The data on exposure history were collected on the basis of in-depth epidemiological investigations, allowing us to provide a fitting estimation of the distribution of the time intervals of key transmission events. We noted that there was considerable uncertainty in the previous estimates generated prior to the emergence of SARS-CoV-2 variants of concern, and previous studies reported ranges from 3.0 to 7.8 days for the SI (33–39) and from 4.8 to 8.0 days for the incubation period (33, 40–45). In this study, we observed that the mean SI was 3.2 days for the BA.2 variant. Moreover, the mean incubation period estimates were shorter (2.4 days) than previous estimates for BA.1 (3.2–4.6 days) (9), BA.2 (4.4 days) (10), and BA.5 (5.7 days) (16). Only three studies (10, 16, 33) provided GI estimates because it was difficult to obtain the required information on the infection dates of both the index cases and their close contacts. We found a mean GI of 2.7 days, which was shorter than those of the previous Alpha (4.7 days) and Delta (5.5 days) variants (33), but longer than that of the Omicron BA.1 variant (2.4 days) (10). Moreover, it was slightly shorter than that of the Omicron BA.5 variant (2.8 days) (16). Finally, we found that the mean latent and viral shedding periods were approximately 2.1 days and 17.9 days, respectively. Previously uncertain estimated time intervals for key events may have resulted from small sample sizes and possible sampling biases (46, 47). Our data were based on an entire epidemic wave and had a larger sample size. These factors may mean that this study does not have the same bias and may provide stable results (47).

In this study, we evaluated the characteristics, dynamics, and risk of Omicron BA.2 transmission based on a major outbreak of the epidemic. It was the first study to comprehensively evaluate Omicron BA.2 sub-lineage SAI and transmission risk, and the first study to assess the effectiveness of inactivated COVID-19 vaccine boosters against the transmission of SARS-CoV-2 Omicron variant in comparison with a full schedule of vaccination without boosters. However, there were also several limitations. First, we used the positive PCR test (Ct value < 40) to estimate the time intervals of key effects. The use of PCR test positivity may impact the accuracy of transmission dynamics assessments due to its imperfect sensitivity caused by the potential delays in viral load reaching detectable levels. However, at present, PCR test is the gold standard for diagnosing SARS-CoV-2 infection. In addition, in our study, all the close contacts received multiple tests during medical observation period, which increased the possibility of early diagnosis of SARS-CoV-2 infection and made up for the low sensitivity of PCR. Second, different countries implemented different measures to control the COVID-19 outbreak. Those different measures may influence the incidence of SAI among close contacts and limit the generalization of our results. Third, our estimates of the high VE for adults of over 60 years old may be associated with a relatively small sample size. Further studies are needed to identify our findings. Fourth, because our data relied on the epidemiological contact-tracing data, we could not always accurately reconstruct the entire transmission chain and fully avoid recall bias.

In summary, the main Omicron BA.2 subvariant transmission setting was in households, and the effectiveness of inactivated vaccine boosters against BA.2 subvariant transmission was relatively high in older people. These findings indicate the importance of continuously assessing VE against different Omicron variant sub-lineages as they quickly evolve and mutate.

## Data availability statement

The original contributions presented in the study are included in the article/**Supplementary Material**. Further inquiries can be directed to the corresponding authors.

## Ethics statement

The study involving human participants were reviewed and approved by the China CDC Ethical Review Committee (approval number 202210). Written informed consent was waived because the data in the study were collected from administrative requirements of disease control and surveillance by Shenzhen Center for Disease Control and Prevention as required by the National Health Commission. Analytical data sets were de-linked and anonymized in this study.

## Author contributions

XH: Conceptualization, Data curation, Formal analysis, Methodology, Project administration, Validation, Writing – original draft, Writing – review & editing. YXL: Conceptualization, Data curation, Investigation, Writing – original draft, Writing – review & editing. YHL: Formal analysis, Writing – review & editing. JY: Writing – review & editing. WG: Data curation, Investigation, Writing – review & editing. JW: Data curation, Investigation, Writing – review & editing. YL: Investigation, Writing – review & editing. JS: Writing – review & editing. XZ: Conceptualization, Investigation, Project administration, Supervision, Writing – original draft, Writing – review & editing. ST: Conceptualization, Project administration, Supervision, Writing – original draft, Writing – review & editing.

## References

[1] WHO. Currently circulating variants of concern (VOCs). World Health Organization (2023). Available at: https://www.who.int/activities/tracking-SARS-CoV-2-variants.

[2] WHO. Weekly epidemiological update on COVID-19-21 September 2022. World Health Organization. Available at: https://www.who.int/publications/m/item/weekly-epidemiological-update-on-covid-19---21-september-2022.

[3] Ren SY Wang WB Gao RD Zhou AM. Omicron variant (B.1.1.529) of SARS-CoV-2: Mutation, infectivity, transmission, and vaccine resistance. World J Clin cases (2022) 10:1–11. doi: 10.12998/wjcc.v10.i1.1 35071500 PMC8727245

[4] Tian D Sun Y Xu H Ye Q. The emergence and epidemic characteristics of the highly mutated SARS-CoV-2 Omicron variant. J Med Virol (2022) 94:2376–83. doi: 10.1002/jmv.27643 PMC9015498 35118687

[5] Kraemer MUG Pybus OG Fraser C Cauchemez S Rambaut A Cowling BJ. Monitoring key epidemiological parameters of SARS-CoV-2 transmission. Nat Med (2021) 27:1854–5. doi: 10.1038/s41591-021-01545-w 34750555

[6] Farahat RA Abdelaal A Umar TP El-Sakka AA Benmelouka AY Albakri K. The emergence of SARS-CoV-2 Omicron subvariants: current situation and future trends. Infez Med (2022) b30:480–94. doi: 10.53854/liim-3004-2 PMC9714996 36482957

[7] Kang M Xin H Yuan J Ali ST Liang Z Zhang J. Transmission dynamics and epidemiological characteristics of SARS-CoV-2 Delta variant infections in Guangdong, China, May to June 2021. Euro Surveill (2022) 27:2100815. doi: 10.2807/1560-7917.ES.2022.27.10.2100815 35272744 PMC8915401

[8] Ganyani T Kremer C Chen D Torneri A Faes C Wallinga J. Estimating the generation interval for coronavirus disease (COVID-19) based on symptom onset data, March 2020. Euro Surveill (2020) 25:12–9. doi: 10.2807/1560-7917.ES.2020.25.17.2000257 PMC7201952 32372755

[9] Backer JA Eggink D Andeweg SP Veldhuijzen IK van Maarseveen N Vermaas K. Shorter serial intervals in SARS-CoV-2 cases with Omicron BA.1 variant compared with Delta variant, the Netherlands, 13 to 26 December 2021. Euro Surveill (2022) 27:2200042. doi: 10.2807/1560-7917.ES.2022.27.6.2200042 35144721 PMC8832521

[10] Mefsin YM Chen D Bond HS Lin Y Cheung JK Wong JY. Epidemiology of infections with SARS-coV-2 omicron BA.2 variant, hong kong, january-march 2022. Emerg Infect Dis (2022) 28:1856–8. doi: 10.3201/eid2809.220613 PMC9423929 35914518

[11] McMenamin ME Nealon J Lin Y Wong JY Cheung JK Lau EHY. Vaccine effectiveness of one, two, and three doses of BNT162b2 and CoronaVac against COVID-19 in Hong Kong: a population-based observational study. Lancet Infect Dis (2022) 22:1435–43. doi: 10.1016/S1473-3099(22)00345-0 PMC9286709 35850128

[12] Huang Z Xu S Liu J Wu L Qiu J Wang N. Effectiveness of inactivated and Ad5-nCoV COVID-19 vaccines against SARS-CoV-2 Omicron BA. 2 variant infection, severe illness, and death. BMC Med (2022) 20:400. doi: 10.1186/s12916-022-02606-8 36266697 PMC9583051

[13] Wan EYF Mok AHY Yan VKC Wang B Zhang R Hong SN. Vaccine effectiveness of BNT162b2 and CoronaVac against SARS-CoV-2 Omicron BA.2 infection, hospitalisation, severe complications, cardiovascular disease and mortality in patients with diabetes mellitus: a case control study. J Infect (2022) 85:e140–4. doi: 10.1016/j.jinf.2022.08.008 PMC9381942 35985416

[14] Cheng FWT Fan M Wong CKH Chui CSL Lai FTT Li X. The effectiveness and safety of mRNA (BNT162b2) and inactivated (CoronaVac) COVID-19 vaccines among individuals with chronic kidney diseases. Kidney Int (2022) 102:922–5. doi: 10.1016/j.kint.2022.07.018 PMC9367175 35964798

[15] Tang L Wang FZ Rodewald LE Wang XY Liu SY Liu QQ. Real-world effectiveness of primary series and booster doses of inactivated COVID-19 vaccine against Omicron BA.2 variant infection in China: a retrospective cohort study. J Infect Dis (2023) 228:261–9. doi: 10.1093/infdis/jiad090 PMC10420401 37005365

[16] Wang K Guo Z Zeng T Sun S Lu Y Wang J. Transmission characteristics and inactivated vaccine effectiveness against transmission of SARS-coV-2 omicron BA.5 variants in urumqi, China. JAMA Netw Open (2023) 6:e235755. doi: 10.1001/jamanetworkopen.2023.5755 36995713 PMC10064257

[17] de Gier B Andeweg S Joosten R Ter Schegget R Smorenburg N van de Kassteele J. Vaccine effectiveness against SARS-CoV-2 transmission and infections among household and other close contacts of confirmed cases, the Netherlands, February to May 2021. Euro Surveill (2021) 26:2100640. doi: 10.2807/1560-7917.ES.2021.26.31.2100640 34355689 PMC8343550

[18] National Health Commission of the People’s Republic of China. The prevention and Control Scheme of COVID-19. Available at: https://www.nhc.gov.cn/jkj/s3577/202105/6f1e8ec6c4a540d99fafef52fc86d0f8/files/4a860a7e85d14d55a22fbab0bbe77cd9.pdf. 10.46234/ccdcw2020.082 PMC8392946 34594648

[19] Hu S Wang W Wang Y Litvinova M Luo K Ren L. Infectivity, susceptibility, and risk factors associated with SARS-CoV-2 transmission under intensive contact tracing in Hunan, China. Nat Commun (2021) 12:1533. doi: 10.1038/s41467-021-21710-6 33750783 PMC7943579

[20] Madewell ZJ Yang Y Longini IM Jr Halloran ME Dean NE. Household secondary attack rates of SARS-CoV-2 by variant and vaccination status: an updated systematic review and meta-analysis. JAMA Netw Open (2022) 5:e229317. doi: 10.1001/jamanetworkopen.2022.9317 35482308 PMC9051991

[21] Zhao S Chong MKC Ryu S Guo Z He M Chen B. Characterizing superspreading potential of infectious disease: decomposition of individual transmissibility. PloS Comput Biol (2022) 18:e1010281. doi: 10.1371/journal.pcbi.1010281 35759509 PMC9269899

[22] Nishiura H Yan P Sleeman CK Mode CJ. Estimating the transmission potential of supercritical processes based on the final size distribution of minor outbreaks. J Theor Biol (2012) 294:48–55. doi: 10.1016/j.jtbi.2011.10.039 22079419 PMC3249525

[23] Jackson ML Nelson JC. The test-negative design for estimating influenza vaccine effectiveness. Vaccine (2013) 31:2165–8. doi: 10.1016/j.vaccine.2013.02.053 23499601

[24] Bond HS Sullivan SG Cowling BJ. Regression approaches in the test-negative study design for assessment of influenza vaccine effectiveness. Epidemiol Infect (2016) 144:1601–11. doi: 10.1017/S095026881500309X PMC5545127 26732691

[25] Cowling BJ Perera RA Fang VJ Chan KH Wai W So HC. Incidence of influenza virus infections in children in Hong Kong in a 3-year randomized placebo-controlled vaccine study, 2009-2012. Clin Infect Dis (2014) 59:517–24. doi: 10.1093/cid/ciu356 24825868

[26] Lopez Bernal J Andrews N Gower C Robertson C Stowe J Tessier E. Effectiveness of the Pfizer-BioNTech and Oxford-AstraZeneca vaccines on covid-19 related symptoms, hospital admissions, and mortality in older adults in England: test negative case-control study. BMJ (2021) 373:n1088. doi: 10.1136/bmj.n1088 33985964 PMC8116636

[27] Andrews N Stowe J Kirsebom F Toffa S Rickeard T Gallagher E. Covid-19 vaccine effectiveness against the omicron (B.1.1.529) variant. N Engl J Med (2022) 386:1532–46. doi: 10.1056/NEJMoa2119451 PMC8908811 35249272

[28] Zeng B Gao L Zhou Q Yu K Sun F. Effectiveness of COVID-19 vaccines against SARS-CoV-2 variants of concern: a systematic review and meta-analysis. BMC Med (2022) 20:200. doi: 10.1186/s12916-022-02397-y 35606843 PMC9126103

[29] Sacco C Del Manso M Mateo-Urdiales A Rota MC Petrone D Riccardo F. Effectiveness of BNT162b2 vaccine against SARS-CoV-2 infection and severe COVID-19 in children aged 5-11 years in Italy: a retrospective analysis of January-April, 2022. Lancet (2022) 400:97–103. doi: 10.1016/S0140-6736(22)01185-0 35780801 PMC9246475

[30] Tseng HF Ackerson BK Luo Y Sy LS Talarico CA Tian Y. Effectiveness of mRNA-1273 against SARS-CoV-2 Omicron and Delta variants. Nat Med (2022) 28(5):1063–71. doi: 10.1038/s41591-022-01753-y PMC9117141 35189624

[31] McMenamin ME Nealon J Lin Y Wong JY Cheung JK Lau EHY. Vaccine effectiveness of one, two, and three doses of BNT162b2 and CoronaVac against COVID-19 in Hong Kong: a population-based observational study. Lancet Infect Dis (2022) 22:1435–43. doi: 10.1016/S1473-3099(22)00345-0 PMC9286709 35850128

[32] de Gier B Andeweg S Backer JA. RIVM COVID-19 surveillance and epidemiology team; Hahné SJ, van den Hof S, et al. Vaccine effectiveness against SARS-CoV-2 transmission to household contacts during dominance of Delta variant (B.1.617.2), the Netherlands, August to September 2021. Euro Surveill (2021). 26 :2100977. doi: 10.2807/1560-7917.ES.2021.26.44.2100977 PMC8569927 34738514

[33] Li Q Guan X Wu P Wang X Zhou L Tong Y. Early transmission dynamics in Wuhan, China, of novel coronavirus–infected pneumonia. N Engl J Med (2020) 382:1199–207. doi: 10.1056/NEJMoa2001316 PMC7121484 31995857

[34] Du Z Xu X Wu Y Wang L Cowling BJ Meyers LA. Serial interval of COVID-19 among publicly reported confirmed cases. Emerg Infect Dis (2020) 26:1341–3. doi: 10.3201/eid2606.200357 PMC7258488 32191173

[35] Nishiura H Linton NM Akhmetzhanov AR. Serial interval of novel coronavirus (COVID-19) infections. Int J Infect Dis (2020) 93:284–6. doi: 10.1016/j.ijid.2020.02.060 PMC7128842 32145466

[36] Danis K Epaulard O Bénet T Gaymard A Campoy S Botelho-Nevers E. Cluster of coronavirus disease 2019 (COVID-19) in the French Alps, February 2020. Clin Infect Dis (2020) 71:825–32. doi: 10.1093/cid/ciaa424 PMC7184384 32277759

[37] He X Lau EHY Wu P Deng X Wang J Hao X. Temporal dynamics in viral shedding and transmissibility of COVID-19. Nat Med (2020) 26:672–5. doi: 10.1038/s41591-020-0869-5 32296168

[38] Prete CA Buss L Dighe A Porto VB da Silva Candido D Ghilardi F. Serial interval distribution of SARS-CoV-2 infection in Brazil. J Travel Med (2021) 28:taaa115. doi: 10.1093/jtm/taaa115 32710618 PMC7454808

[39] You C Deng Y Hu W Sun J Lin Q Zhou F. Estimation of the time-varying reproduction number of COVID19 outbreak in China. Int J Hyg Environ Health (2020) 228:113555. doi: 10.1016/j.ijheh.2020.113555 32460229 PMC7211652

[40] Zhang J Litvinova M Wang W Wang Y Deng X Chen X. Evolving epidemiology and transmission dynamics of coronavirus disease 2019 outside Hubei province, China: a descriptive and modelling study. Lancet Infect Dis (2020) 20:793–802. doi: 10.1016/S14733099(20)30230-9 32247326 PMC7269887

[41] Linton NM Kobayashi T Yang Y Hayashi K Akhmetzhanov AR Jung SM. Incubation period and other epidemiological characteristics of 2019 novel coronavirus infections with right truncation: a statistical analysis of publicly available case data. J Clin Med (2020) 9:538. doi: 10.3390/jcm9020538 32079150 PMC7074197

[42] Backer JA Klinkenberg D Wallinga J. Incubation period of 2019 novel coronavirus (2019-nCoV) infections among travellers from Wuhan, China, 20–28 January 2020. Euro Surveill (2020) 25:2000062. doi: 10.2807/1560-7917.ES.2020.25.5.2000062 32046819 PMC7014672

[43] Bi Q Wu Y Mei S Ye C Zou X Zhang Z. Epidemiology and transmission of COVID-19 in 391 cases and 1286 of their close contacts in Shenzhen, China: a retrospective cohort study. Lancet Infect Dis (2020) 20:911–9. doi: 10.1016/S1473-3099(20)30287-5 PMC7185944 32353347

[44] Tian S Hu N Lou J Chen K Kang X Xiang Z. Characteristics of COVID-19 infection in beijing. J Infect (2020) 80:401–6. doi: 10.1016/j.jinf.2020.02.018 PMC7102527 32112886

[45] Xu T Chen C Zhu Z Cui M Chen C Dai H. Clinical features and dynamics of viral load in imported and non-imported patients with COVID-19. Int J Infect Dis (2020) 94:68–71. doi: 10.1016/j.ijid.2020.03.022 32179140 PMC7270709

[46] Nishiura H Linton NM Akhmetzhanov AR. Serial interval of novel coronavirus (COVID-19) infections. Int J Infect Dis (2020) 93:284–6. doi: 10.1016/j.ijid.2020.02.060 PMC7128842 32145466

[47] Park SW Sun K Champredon D Li M Bolker BM Earn DJD. Forward-looking serial intervals correctly link epidemic growth to reproduction numbers. Proc Natl Acad Sci USA (2021) 118:e2011548118. doi: 10.1073/pnas.2011548118 33361331 PMC7812760

