# Supplementary material for: Transmission characteristics and inactivated vaccine effectiveness against transmission of the SARS-CoV-2 Omicron BA.2 variant in Shenzhen, China
Source: Front Immunol. 2024 Jan 8;14:1290279. doi: 10.3389/fimmu.2023.1290279 (PMC10800792; doi:10.3389/fimmu.2023.1290279)
Supplement: Supplementary file 1 [file Table_1.docx]

**S1 Table** Characteristics of SARS-CoV-2 BA.2 infections, Shenzhen, China, February–April 2022

| **Characteristics** | **Asymptomatic infections**  **(N = 203 [16.3])** | **Mild COVID-19**  **(N = 975 [78.1])** | **COVID-19 pneumonia**  **(N = 70 [5.6])** | ***P* value** | **Overall**  **N = 1248** |
| --- | --- | --- | --- | --- | --- |
| **Age, years** | | | | | |
| Median, IQR | 37 (29, 50) | 34 (25, 46) | 37.5 (27.8, 48.3) | 0.016 | 35 (25, 47) |
| 0-9 | 14 (6.9) | 94 (9.6) | 7 (10.0) | 0.256 | 115 (9.2) |
| 10-19 | 8 (3.9) | 68 (7.0) | 4 (5.7) |  | 80 (6.4) |
| 20-29 | 32 (15.8) | 201 (20.6) | 10 (14.3) |  | 243 (19.5) |
| 30-39 | 59 (29.1) | 255 (26.2) | 16 (22.9) |  | 330 (26.4) |
| 40-49 | 37 (18.2) | 174 (17.8) | 17 (24.2) |  | 228 (18.3) |
| 50-59 | 38 (18.7) | 124 (12.7) | 10 (14.3) |  | 172 (13.8) |
| ≥ 60 | 15 (7.4) | 59 (6.1) | 6 (8.6) |  | 80 (6.4) |
| **Gender** | | | | | |
| Male | 124 (61.1) | 547 (56.1) | 39 (55.7) | 0.419 | 710 (56.9) |
| Female | 79 (39.9) | 428 (43.9) | 31 (44.3) |  | 538 (43.1) |
| **COVID-19 vaccination status*** | | | | | |
| None | 23 (11.3) | 118 (12.1) | 8 (11.4) | 0.913 | 149 (11.9) |
| Partial vaccination | 9 (4.5) | 43 (4.4) | 1 (1.4) |  | 53 (4.3) |
| Full vaccination | 78 (38.4) | 394 (40.4) | 30 (42.9) |  | 502 (40.2) |
| Booster vaccination | 93 (45.8) | 420 (43.1) | 31 (44.3) |  | 544 (43.6) |
| **COVID-19 vaccines type** | | | | | |
| Non-inactivated | 5 (2.5) | 16 (1.6) | 1 (1.4) | 0.731 | 22 (1.8) |
| Inactivated vaccines | 175 (86.2) | 841 (86.3) | 61 (87.1) |  | 1077 (97.2) |

*None: not vaccinated; partial vaccination: < 14 days after first vaccination for viral vector (non-replicating) vaccine, after first vaccination or < 14 days after second vaccination for COVID-19 inactivated virus vaccine, and after first and second vaccination, or < 14 days after third vaccination COVID-19 protein subunit vaccine (if any); full vaccination: ≥ 14 days after first vaccination for viral vector (non-replicating) vaccine, ≥ 14 days after second vaccination for COVID-19 inactivated virus vaccine, ≥ 14 days after third vaccination for COVID-19 protein subunit vaccine, and < 7 days after booster vaccination (if any); booster vaccination: ≥ 7 days after second dose for COVID-19 viral vector (non-replicating) vaccines or ≥ 7 days after third dose for COVID-19 any vaccine (including protein subunit, inactivated virus, and viral vector [non-replicating] vaccines) (if any).
