# Supplementary material for: Transmission characteristics and inactivated vaccine effectiveness against transmission of the SARS-CoV-2 Omicron BA.2 variant in Shenzhen, China
Source: Front Immunol. 2024 Jan 8;14:1290279. doi: 10.3389/fimmu.2023.1290279 (PMC10800792; doi:10.3389/fimmu.2023.1290279)
Supplement: Supplementary file 2 [file Table_2.docx]

**S2 Table** The main clinical symptoms characteristics of 947 infections with symptomatic COVID-19 by Vaccination Status*

| **Characteristic** | **None/** **Partial vaccination**  **(N = 157)** | **Full vaccination**  **(N = 382)** | **Booster vaccination**  **(N = 408)** | ***P* value** | **Overall**  **(N = 947)** |
| --- | --- | --- | --- | --- | --- |
| Pyrexia | 121 (77.1) | 267 (69.9) | 185 (45.3) | < 0.001 | 573 (60.5) |
| Cough | 43 (27.4) | 128 (33.5) | 170 (41.7) | 0.003 | 341 (36.0) |
| Pharyngeal pain | 24 (15.3) | 77 (20.2) | 122 (29.9) | < 0.001 | 223 (23.5) |
| Fatigue | 14 (8.9) | 69 (18.1) | 52 (12.7) | 0.011 | 135 (14.3) |
| R[hinorrhea](javascript:;) | 8 (5.1) | 18 (4.7) | 29 (6.9) | 0.326 | 55 (5.8) |
| Nasal congestion | 6 (3.8) | 18 (4.7) | 31 (7.6) | 0.113 | 55 (5.8) |
| Myalgia | 3 (1.9) | 22 (5.8) | 19 (4.7) | 0.156 | 44 (4.7) |
| Throat-drying | 7 (4.5) | 14 (3.7) | 19 (4.7) | 0.777 | 40 (4.2) |
| Diarrhea | 5 (3.2) | 10 (2.6) | 16 (3.9) | 0.587 | 31 (3.3) |
| Pharyngeal itching | 2 (1.3) | 6 (1.6) | 17 (4.2) | 0.038 | 25 (2.6) |
| Anosmia/Ageusia | 2 (1.3) | 8 (2.1) | 8 (2.0) | 0.812 | 18 (1.9) |

Data are n (%), unless otherwise specified. *None: not vaccinated; partial vaccination: < 14 days after first vaccination for viral vector (non-replicating) vaccine, after first vaccination or < 14 days after second vaccination for COVID-19 inactivated virus vaccine, and after first and second vaccination, or < 14 days after third vaccination COVID-19 protein subunit vaccine (if any); full vaccination: ≥ 14 days after first vaccination for viral vector (non-replicating) vaccine, ≥ 14 days after second vaccination for COVID-19 inactivated virus vaccine, ≥ 14 days after third vaccination for COVID-19 protein subunit vaccine, and < 7 days after booster vaccination (if any); booster vaccination: ≥ 7 days after second dose for COVID-19 viral vector (non-replicating) vaccines or ≥ 7 days after third dose for COVID-19 any vaccine (including protein subunit, inactivated virus, and viral vector [non-replicating] vaccines) (if any).
