# Supplementary material for: Transmission characteristics and inactivated vaccine effectiveness against transmission of the SARS-CoV-2 Omicron BA.2 variant in Shenzhen, China
Source: Front Immunol. 2024 Jan 8;14:1290279. doi: 10.3389/fimmu.2023.1290279 (PMC10800792; doi:10.3389/fimmu.2023.1290279)
Supplement: Supplementary file 3 [file Table_3.docx]

**S3 Table** SAR and SCT Summary

| **Stratification** | **Index cases** | **Close contacts** | **Secondary Cases** | **SAR**  **(%, [95% CI])** | **SCT**  **(%, [95% CI]) ^#^** |
| --- | --- | --- | --- | --- | --- |
| **Overall** | 644 | 8466 | 611 | 7.2 (6.7, 7.8) | 13.5 (11.4, 16.0) |
| **Age (years)** | | | | | |
| 0-17 | 63 | 608 | 110 | 18.1 (15.2, 21.4) | 11.5 (6.5, 19.4) |
| 18-59 | 548 | 7493 | 452 | 6.0 (5.5, 6.6) | 13.0 (10.7, 15.7) |
| ≥ 60 | 33 | 365 | 49 | 13.4 (10.3, 17.3) | 26.8 (15.7, 41.9) |
| **Gender** | | | | | |
| Male | 368 | 5150 | 328 | 6.4 (5.7, 7.1) | 11.7 (9.2, 14.7) |
| Female | 276 | 3316 | 283 | 8.5 (7.6, 9.5) | 16.3 (12.8, 20.6) |
| **COVID-19 vaccine dose*** | | | | | |
| None/partial vaccination | 95 | 928 | 102 | 11.0 (9.1, 13.2) | 16.7 (11.2, 24.1) |
| Full vaccination | 232 | 3020 | 237 | 7.9 (6.9, 8.9) | 16.5 (9.2, 16.7) |
| Booster vaccination | 317 | 4518 | 272 | 6.0 (5.4, 6.8) | 13.3 (10.4, 17.0) |
| **Type of cases** | | | | | |
| Asymptomatic infection | 101 | 1521 | 105 | 6.9 (5.7, 8.3) | 14.3 (9.5, 20.9) |
| Symptomatic COVID-19 | 543 | 6945 | 506 | 7.3 (6.7, 7.9) | 13.3 (11.0, 16.0) |

SAR, secondary attack rate; SCT, supercritical transmission.

*None: not vaccinated; partial vaccination: < 14 days after first vaccination for viral vector (non-replicating) vaccine, after first vaccination or < 14 days after second vaccination for COVID-19 inactivated virus vaccine, and after first and second vaccination, or < 14 days after third vaccination COVID-19 protein subunit vaccine (if any); full vaccination: ≥ 14 days after first vaccination for viral vector (non-replicating) vaccine, ≥ 14 days after second vaccination for COVID-19 inactivated virus vaccine, ≥ 14 days after third vaccination for COVID-19 protein subunit vaccine, and < 7 days after booster vaccination (if any); booster vaccination: ≥ 7 days after second dose for COVID-19 viral vector (non-replicating) vaccines or ≥ 7 days after third dose for COVID-19 any vaccine (including protein subunit, inactivated virus, and viral vector [non-replicating] vaccines) (if any). ^#^ For the calculation of SCT proportion, all 1248 SARS-CoV-2 infections were involved because the number of offspring infections associated with each index infection was known.
