# Supplementary material for: Transmission characteristics and inactivated vaccine effectiveness against transmission of the SARS-CoV-2 Omicron BA.2 variant in Shenzhen, China
Source: Front Immunol. 2024 Jan 8;14:1290279. doi: 10.3389/fimmu.2023.1290279 (PMC10800792; doi:10.3389/fimmu.2023.1290279)
Supplement: Supplementary file 4 [file Table_4.docx]

**S4 Table** Estimated Mean of Key Time Intervals for Transmission of SARS-CoV-2 Omicron BA.2 Variants

| **Time interval** | **Stratification** | **Sample size** | **Parameters [mean (SD)]** | **Mean (days)** | **Quantiles (0.025, 0.975; days)** |
| --- | --- | --- | --- | --- | --- |
| Generation Interval | **Overall** | 604 | Shape = 79.19 (4.55), rate = 4.6 (0.26), shift = 14.5 | 2.7 | –0.8, 6.7 |
|  | **Contact setting** | | | | |
|  | Household | 243 | Shape = 92.72 (8.4), rate = 5.49 (0.5), shift = 14.5 | 2.4 | –0.9, 6.0 |
|  | Non-household | 361 | Shape = 74.28 (5.52), rate = 4.27 (0.32), shift = 14.5 | 2.9 | –0.8, 7.0 |
| Serial Interval | Overall | 419 | Shape =73.45 (5.06), rate = 4.15 (0.29), shift = 14.5 | 3.2 | –0.6, 7.4 |
|  | **Contact setting** | | | | |
|  | Household | 170 | Shape = 80.16 (8.68), rate = 4.54 (0.49), shift = 14.5 | 3.2 | –0.5, 7.2 |
|  | Non-household | 249 | Shape = 69.46 (6.21), rate = 3.92 (0.35), shift = 14.5 | 3.2 | –0.7, 7.6 |
| Period  from exposure  to viral shedding (Latent period) | Overall | 394 | Shape = 0.55 (0.04), rate = 0.26 (0.02) | 2.1 | 0.0, 9.7 |
|  | **Age group** | | | | |
|  | 0-17 | 80 | Shape = 0.30 (0.05), rate = 0.19(0.05) | 1.5 | 0.0, 9.7 |
|  | 18-59 | 282 | Shape = 0.74 (0.06), rate = 0.33(0.03) | 2.2 | 0.0, 9.4 |
|  | ≥ 60 | 32 | Shape = 0.35 (0.09), rate = 0.17(0.07) | 2.0 | 0.0, 11.4 |
|  | **Symptom status** | | | | |
|  | Asymptomatic | 72 | Shape = 0.58 (0.09), rate = 0.29(0.06) | 2.0 | 0.0, 9.1 |
|  | Symptomatic | 322 | Shape = 0.54 (0.04), rate = 0.25(0.03) | 2.1 | 0.0, 9.9 |
|  | **COVID-19 vaccination status** | | | | |
|  | None or partial | 67 | Shape = 0.36 (0.06), rate = 0.17 (0.05) | 2.0 | 0.0, 11.6 |
|  | Full | 162 | Shape = 0.44 (0.05), rate = 0.22 (0.04) | 1.9 | 0.0, 10.1 |
|  | Booster | 165 | Shape = 0.89 (0.09), rate = 0.40 (0.05) | 2.2 | 0.0, 8.6 |
| Viral  shedding  period | Overall | 1244 | Shape = 12.55 (0.50), rate = 0.71 (0.03) | 17.9 | 9.4, 28.5 |
|  | **Age group** | | | | |
|  | 0-17 | 182 | Shape = 12.58 (1.30), rate = 0.75 (0.08) | 16.7 | 8.9, 27.0 |
|  | 18-59 | 982 | Shape = 12.49 (0.56), rate = 0.70 (0.03) | 17.9 | 9.5, 29.0 |
|  | ≥ 60 | 80 | Shape = 15.40 (2.41), rate = 0.83 (0.13) | 18.6 | 10.6, 28.8 |
|  | **Symptom status** | | | | |
|  | Asymptomatic | 203 | Shape = 9.67 (0.94), rate = 0.57 (0.06) | 17.1 | 8.2, 29.4 |
|  | Symptomatic | 1041 | Shape = 13.38 (0.58), rate = 0.75 (0.03) | 17.9 | 9.8, 28.6 |
|  | **COVID-19 vaccination status** | | | | |
|  | None or partial | 200 | Shape = 14.84 (1.47), rate = 0.81 (0.08) | 18.3 | 10.3 - 28.5 |
|  | Full | 487 | Shape = 11.35 (0.72), rate = 0.63 (0.04) | 18.0 | 9.2, 29.7 |
|  | Booster | 557 | Shape = 12.55 (0.50), rate = 0.71 (0.03) | 17.8 | 9.5, 28.7 |
| Incubation period | Overall | 323 | Shape = 0.75 (0.06), rate = 0.31 (0.03) | 2.4 | 0.0, 9.9 |
|  | **Age** | | | | |
|  | 0-17 | 71 | Shape = 0.53 (0.08), rate = 0.27 (0.06) | 1.9 | 0.0, 9.2 |
|  | 18-59 | 226 | Shape = 1.01 (0.09), rate = 0.39 (0.04) | 2.6 | 0.1, 9.4 |
|  | ≥ 60 | 26 | Shape = 0.32 (0.09), rate = 0.15 (0.07) | 2.0 | 0.0, 12.5 |
|  | **COVID-19 vaccination status** | | | | |
|  | None or partial | 55 | Shape = 0.53 (0.09), rate = 0.22 (0.06) | 2.4 | 0.0, 11.5 |
|  | Full | 139 | Shape = 0.62 (0.07), rate = 0.28 (0.04) | 2.2 | 0.0, 10.0 |
|  | Booster | 129 | Shape = 1.22 (0.14), rate = 0.48 (0.07) | 2.5 | 0.1, 8.6 |
