# Supplementary figures and images for: Transmission characteristics and inactivated vaccine effectiveness against transmission of the SARS-CoV-2 Omicron BA.2 variant in Shenzhen, China
Source: Front Immunol. 2024 Jan 8;14:1290279. doi: 10.3389/fimmu.2023.1290279 (PMC10800792; doi:10.3389/fimmu.2023.1290279)

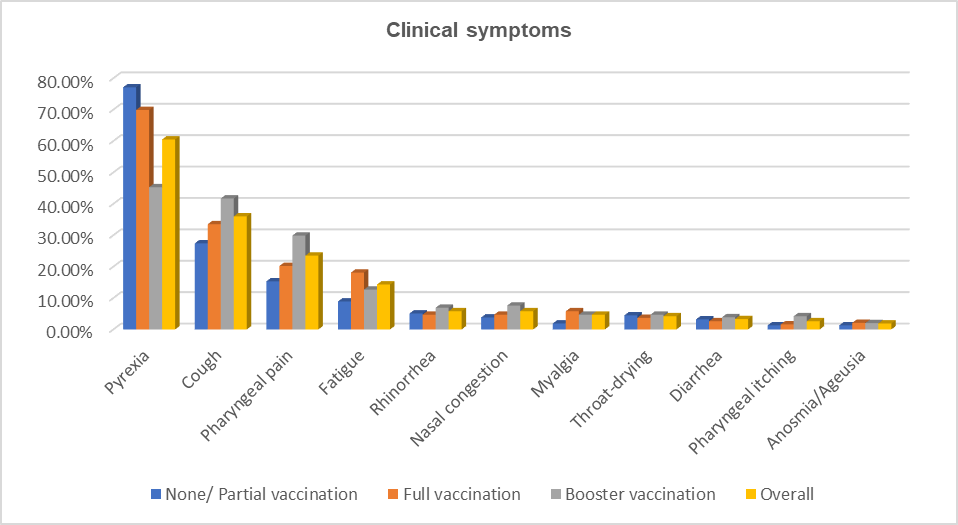


**S1 Figure** The main clinical symptoms characteristics by Vaccination Status

Supplement: Supplementary file 5 [file DataSheet_1.docx]

**
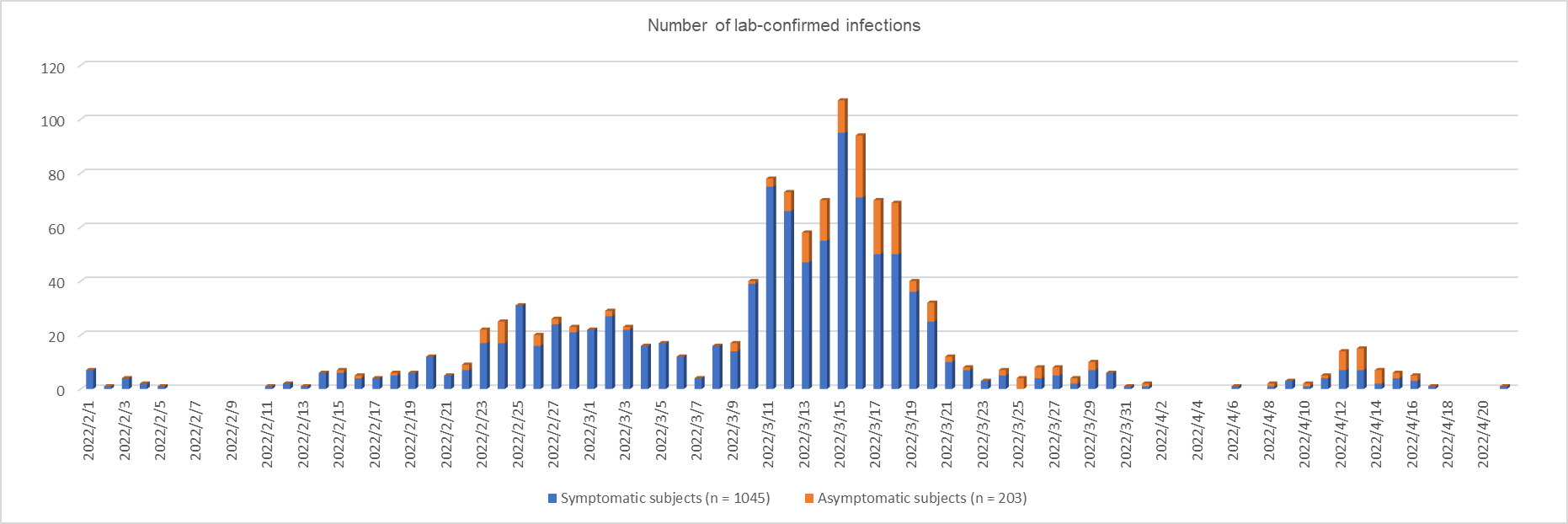
**

**S2 Figure** Date of symptomatic and asymptomatic onset

Supplement: Supplementary file 6 [file DataSheet_2.docx]
