# Supplementary material for: Transmission characteristics and inactivated vaccine effectiveness against transmission of the SARS-CoV-2 Omicron BA.2 variant in Shenzhen, China
Source: Front Immunol. 2024 Jan 8;14:1290279. doi: 10.3389/fimmu.2023.1290279 (PMC10800792; doi:10.3389/fimmu.2023.1290279)
Supplement: Supplementary file 7 [file DataSheet_3.docx]

**S3 Figure** SARS-CoV-2 Omicron BA.2 secondary attack and spreading infection rate by age (years)
